# 4088 Longitudinal Assessment of Metabolic Syndrome as a Modifiable Risk factor of World Trade Center Particulate Matter Exposure Associated Lung Disease

**DOI:** 10.1017/cts.2020.180

**Published:** 2020-07-29

**Authors:** Sophia Kwon, Myeonggyun Lee, Theresa Schwartz, Rachel Zeig-Owens, David Prezant, Mengling Liu, Anna Nolan

**Affiliations:** 1 NYU School of Medicine

## Abstract

OBJECTIVES/GOALS: Metabolic syndrome (MetSyn) is a risk for World Trade Center-Lung Injury (WTC-LI; defined as developing FEV^1^<lower limit of normal [LLN]). Metabolic health is a modifiable disease risk factor. We propose to characterize how time-dependent covariates of MetSyn are longitudinally associated with WTC-LI. METHODS/STUDY POPULATION: WTC-particulate exposed firefighters, consented, with pre-9/11 FEV_1_ LLN (N = 5,746). Data assessed from last pre-9/11 till August 1, 2017. Longitudinal MetSyn characteristics were assessed using 3 models: ***i.*** A linear mixed effect model to assess the effect size of longitudinal MetSyn and its components on longitudinal FEV_1_% predicted as an outcome; ***ii.*** a time-dependent Cox regression to assess the associations of MetSyn to time of onset of WTC-LI; iii. a novel, partially linear single index regression model with repeatedly measured MetSyn to assess their joint effects and delineate their relative contribution on the longitudinal lung function in the WTC-FDNY cohort. RESULTS/ANTICIPATED RESULTS: In **Model I**, BMI 30 kg/m^2^ had the largest effect size compared to ever-smoking, with −2.524 (95%CI: −2.708,−2.340) compared to −1.681(−2.325,−1.038) respectively. Having MetSyn, defined as 3/5 risk factors, had an effect size of −2.319(−2.526,−2.112). In **Model II**, hazards of triglycerides 150mg/dL were highest at 1.497(1.336, 1.677), followed by BMI 30 kg/m^2^ at 1.406(1.256, 1.575), and HDL<40mg/dL 1.355(1.176-1.561), compared to ever-smoking (1.201, p = 0.002). Having high exposure to PM by being present in the morning of 9/11 was a significant covariate only in Model II investigating HDL<40mg/dL or triglycerides 150mg/dL. **Model III** The proposed methods will be applied to our cohort study. DISCUSSION/SIGNIFICANCE OF IMPACT: MetSyn is both a predictor and concurrent marker of WTC-LI. The single index model can not only reduce dimensionality of the covariates, but also provides efficient estimates of the joint MetSyn effects, allowing linear or nonlinear effects. Future studies will investigate dietary intervention as a potential disease-modifying factor. CONFLICT OF INTEREST DESCRIPTION: NA, nothing to disclose.

